# Impact of colonoscopic screening in Familial Colorectal Cancer Type X

**DOI:** 10.1002/mgg3.478

**Published:** 2018-10-09

**Authors:** Elizabeth Hatfield, Jane S. Green, Michael O. Woods, Geoff Warden, Patrick S. Parfrey

**Affiliations:** ^1^ Clinical Epidemiology Unit Memorial University St. John’s Newfoundland Canada; ^2^ Discipline of Genetics, Faculty of Medicine Memorial University St. John’s Newfoundland Canada

**Keywords:** colorectal cancer, familial colorectal cancer type‐X, hereditary non‐polyposis colorectal cancer, incidence, screening, survival

## Abstract

**Background:**

Hereditary Non‐Polyposis Colorectal cancer is caused by Lynch Syndrome (LS; an autosomal dominant condition) or by Familial Colorectal Cancer Type‐X (FCCTX; a condition of high family risk that fulfills Amsterdam criteria). The lifetime risk of developing colorectal cancer (CRC) in FCCTX family members is high and CRC occurs later than in LS.

**Methods:**

To determine the impact of primary prevention colonoscopic screening in asymptomatic first‐degree relatives of incident CRC cases in 20 families with FCCTX, we compared cancer incidence and survival in 79 males and 83 females, assumed to be at 50% risk of inheriting a genetic CRC susceptibility factor, who entered screening to an unscreened control group from the families, matched for age at entry into screening and for sex.

**Results:**

In males, median age at entry into screening was 44.8 years, median follow‐up 12.4 years, 12% developed CRC, and 46% died after 30 years of follow‐up. Compared to the unscreened group, relative risk of CRC was 0.27 (95% confidence intervals (CI) 0.10–0.71). In screened females, comparable results were 44.5 years at entry, 11.2 years of follow‐up, 7.1% developed CRC, and 7.2% died after 30 years of follow‐up. The relative risk of CRC compared to the unscreened group was 0.19 (95% CI 0.07–0.48).

**Conclusion:**

Primary prevention screening colonoscopy in asymptomatic family members significantly decreased the risk of CRC in FCCTX.

## 1 INTRODUCTION

In Canada, colorectal cancer (CRC) is the second leading cancer occurring in males and third in females, with lifetime probabilities of developing CRC at 7.4% and 6.4%, respectively (Canadian Cancer Society, 2017).

Hereditary non‐polyposis colorectal cancer (HNPCC) is an important cause of familial CRC as it is caused by Lynch Syndrome (LS; an autosomal dominant condition) or by Familial Colorectal Cancer Type X (FCCTX; a condition of high family risk that fulfills Amsterdam criteria; (Lindor, 2009). It affects approximately 3%–5% of those with colon cancer (Lindor et al., 2005; Woods et al., 2010). LS tumors are characterized by genomewide microsatellite instability (MSI; Parsons, Li, & Longley, 1993) which is a marker for germline mutations in the mismatch repair (MMR) genes; *MLH1* (Bronner et al., 1994), *MSH2* (Leach, Nicolaides, & Papadopoulos, 1993), *MSH6* (Miyaki et al., 1997), and *PMS2* (Nicolaides, Papadopoulos, & Liu, 1994). Other mutations associated with LS include an *EPCAM* deletion which silences *MSH2* expression (Ligtenberg et al., 2009) and, a mono‐allelic *MLH1* epimutation (Pritchard et al., 2012). FCCTX is a familial CRC syndrome that fulfills Amsterdam Criteria 1, suggesting autosomal dominant inheritance, tumors are microsatellite stable and affected individuals do not have mutations in the MMR genes (Aaltonen, Johns, Jarvinen, Mecklin, & Houlston, 2007; Lindor et al., 2005).

The Amsterdam I Criteria (AC1) were developed to standardize the inclusion criteria for research studies aimed at defining the etiology and clinical course of HNPCC (Vasen, Mecklin, Khan, & Lynch, 1991). The AC1 criteria require at least three relatives with histologically verified colorectal cancer where, (a) one is a first‐degree relative of the other two; (b) at least two successive generations are affected; (c) at least one of the relatives with CRC has been diagnosed at <50 years of age; and (d) Familial Adenomatous Polyposis has been excluded (Vasen, Watson, Mecklin, & Lynch, 1999).

As CRC develops primarily from adenomas, particularly adenomatous or serrated polyps, interval colonoscopic surveillance in people at high risk of CRC may prevent the onset of CRC by removal of polyps with malignant potential (Zauber et al., 2012). There is considerable evidence of the protective effects of colonoscopy from adenoma cohorts (Brenner, Stock, & Hoffmeister, 2014). In LS families, colonoscopic screening every 1–2 years significantly decreased CRC incidence and mortality in asymptomatic male and female *MSH2* mutation carriers (Stuckless et al., 2012). For FCCTX families, the interval screening recommendations for incident family members and their first‐degree relatives include colonoscopy every 3–5 years, starting 10 years before the earliest age of diagnosis of CRC in the immediate family (Rex et al., 2017). This was due to evidence indicating that FCCTX families have an older mean age of onset of CRC than that observed in LS families and a lower lifetime risk of CRC (Lindor et al., 2005). However, there is no evidence on the effectiveness of this strategy.

In Newfoundland, we studied 20 FCCTX families from a cohort of 66 HNPCC families who met AC1 criteria (Warden et al., 2013). The proband’s CRC were MSS and no mutations were found in the MMR genes. Some asymptomatic first‐degree relatives of incident CRC cases in these families entered a primary prevention colonoscopic screening program and others did not. We compared CRC incidence and mortality in screened family members to unscreened family members matched by sex and age (controls were alive and CRC free at the age the family member entered the screening program). In the study of LS families that we had undertaken *MSH2* mutation carriers were at very high risk of CRC (Stuckless et al., 2012). However, first‐degree relatives of incident CRC cases in FCCTX families were considered to be at <50% risk of CRC, because some members did and others did not inherit predisposing genetic factors to CRC.

## 2 METHODS

This study was approved by the Health Research Ethics Board, Non‐Clinical Trials, Newfoundland and Labrador. This is a family‐based case–control study with families identified from the population of Newfoundland. FCCTX families were identified from population‐based cohorts where incident cases with CRC were recruited into the Newfoundland Familial Colorectal Cancer Registry between 1999 and 2003, or had been referred to the Provincial Medical Genetics Program (Woods et al., 2010).

### 2.1 Study participants

**Figure 1:**
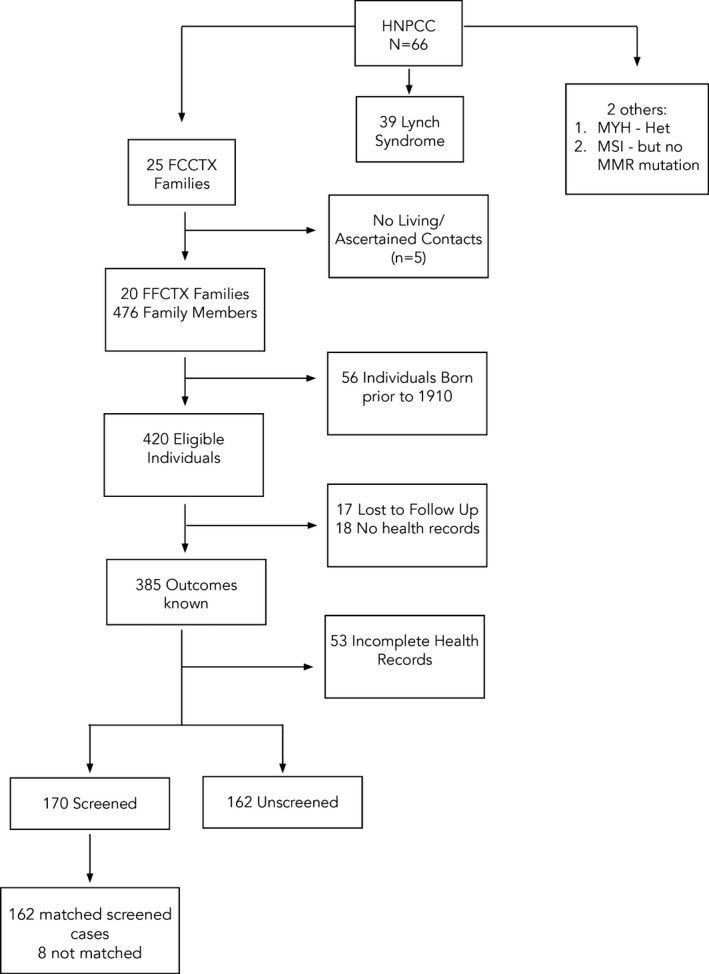
Ascertainment of screened cases and unscreened controls with FCCTX

Of 66 HNPCC families identified in the population‐based cohort, 25 met the criteria for FCCTX: Amsterdam I family history criteria, the proband had a MSS CRC, and no MMR mutation was identified in the proband (Figure 1). Family members eligible for study were born after 1909, were first‐degree relatives of incident CRC cases, and presumed to be at 50% a priori risk for a inheriting genetic CRC susceptibility factor.

Five families had no living/ascertained contacts. From 20 FCCTX families, 420 family members born after 1909 were considered eligible to participate in the study. Of these, 17 individuals were lost to follow‐up, consent for medical records was not received for 18 individuals, and 53 had no/incomplete medical records (Figure 1). Of the remaining 332 first‐degree relatives of incident CRC cases with complete medical records, 170 asymptomatic family members entered a primary prevention colonoscopic screening program and 162 did not. Participants who had entered screening were matched 1:1 to unscreened family members of the same gender, who were cancer free and not in a screening program at the same age as the screened persons. Where several potential controls were available, random matching was undertaken by a third party, blinded to the outcome. One male (71.6 years) and seven females (48.9, 50.7, 50.9, 51.4, 55.6, 68.1, and 78.3 years) could not be matched with controls. The screened group reported here comprised of 83 females and 79 males (Figure 1). CRCs in the screened group occurred despite primary surveillance and CRCs in the unscreened group were detected because they presented with symptoms.

### 2.2 Data collection

Medical records were reviewed, and demographic data, results of genetic testing, diagnosis dates, and details from pathology reports were collected on colorectal cancer, extra‐colonic tumors, and polyps from screening and from operative procedures. Also, data on screening interval recommendations made immediately following colonoscopies were collected from letters of consultation. All data were collected between 2013 and 2016.

Lynch syndrome cancers were defined as those arising from the endometrium, ovary, breast, stomach, ureter or renal pelvis, prostate, bladder, small bowel, hepatobiliary tract, also brain glioblastomas, and sebaceous tumors of the skin (Vasen et al., 1999).

### 2.3 Colonoscopic screening

Prior to the discovery of MMR mutations, asymptomatic members of families with HNPCC assumed to be at 50% risk of acquiring a cancer‐predisposing mutation were recommended to enter a screening colonoscopy program to start at an age 10 years younger than the earliest CRC in the family and to have follow‐up colonoscopies at 1–2‐year intervals. Following the discovery of MMR gene mutations, this program continued in families in whom no MMR gene mutation was identified. Of the 162 FCCTX screened family members reported here, 14 (8.6%) entered the program between 1980 and 1989, 57 (35.2%) entered between 1990 and 1999, and the remaining 91 (56.2%) from 2000 onwards.

### 2.4 Inheritance pattern

For the purpose of this research, three to five generation pedigrees were constructed from the data using Progeny v8 to identify the CRC pattern of inheritance, assess Amsterdam Criteria, and identify first‐degree relatives of incident CRC cases at 50% risk of inheriting genetic CRC susceptibility factor.

### 2.5 Statistical analysis

Statistical analysis was performed using Version 24 of IBM‐ Statistical Package for Social Sciences (SPSS). Cumulative incidences of CRC, extra‐colonic cancers, adenomatous polyps, and all‐cause mortality, were calculated using Kaplan–Meier time‐to‐event analysis both from start of study and from birth. The significance of the difference between the groups was tested using the log rank test. Relative risk of developing each outcome was estimated for the screened group compared to that observed in the unscreened group using the Cox Regression Model. The Pearson chi‐square was used to test for differences in demographic, clinical, and pathology features between groups. To determine whether people entered screening prior to the anticipated time for CRC to occur, we compared age at onset of screening to age at onset of CRC in the unscreened group.

## 3 results

### 3.1 Characteristics of study sample

Of 79 screened asymptomatic males, 67% (*n* = 53) were born after 1950, compared to 34% (*n* = 27) of unscreened males (*p* < 0.001). Of 83 screened asymptomatic females, 71% (*n* = 59) were born after 1950, compared to 24% (*n* = 20) of unscreened females (*p* < 0.001).

**Figure 2:**
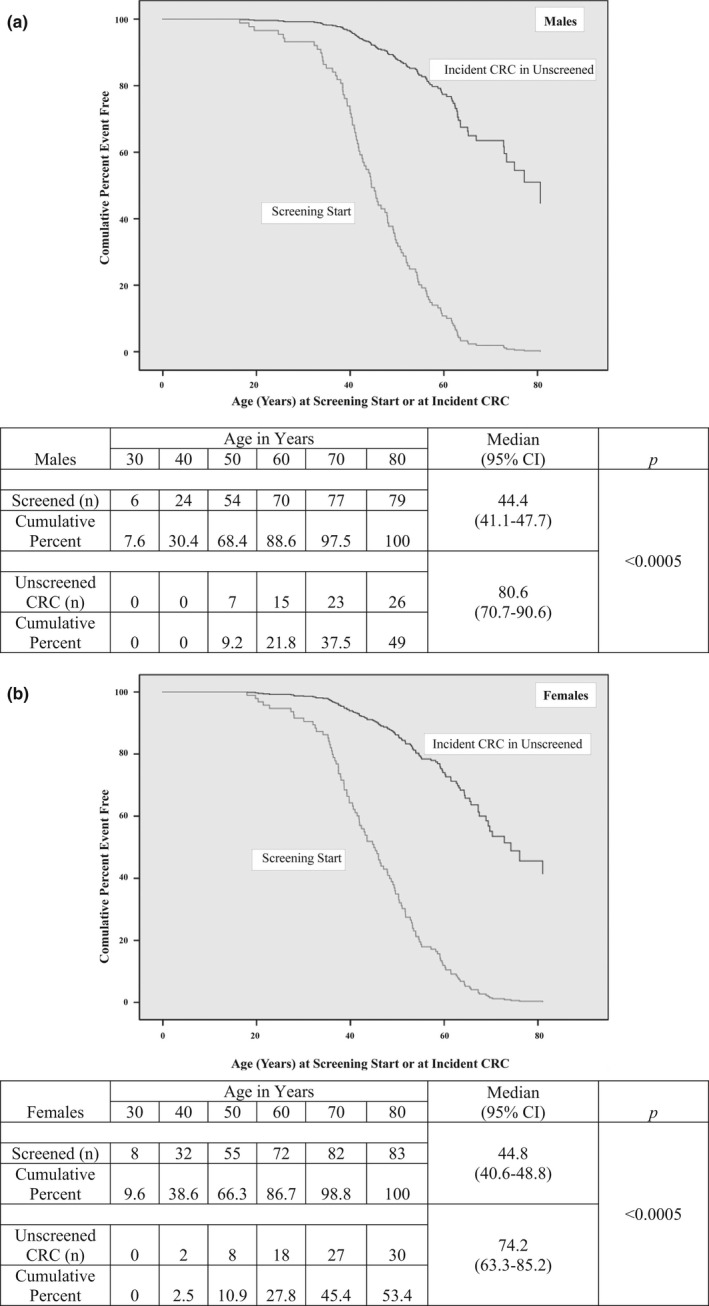
Age at start of screening in the primary prevention group and age at incident CRC in matched unscreened group: (a) Males; (b) Females

The median ages of males and females at entry into screening were similar; 44.8 (95% CI 42.2–47.4) vs. 44.5 (41.8–47.2) years. Median follow‐up time for those in screening was 12.4 (11.5–13.3) years in males, and 11.2 (9.1–13.2) years in females. Comparison of age at onset of screening to age of onset of cancer in the unscreened group is shown in Figure 2. In unscreened males, 9% had developed CRC by age 50 years, whereas 68% of screened males had already entered screening. Comparable proportions for females were 11% and 66%.

#### 3.1.1 Colonoscopy intervals

The mean (± standard deviation) number of colonoscopies in screened males was 7.7 (± 6.0) and in screened females, it was 9.0 (± 5.5). At the time of colonoscopy (initial and follow‐up), physicians made one of five screening interval recommendations (Table 1). The proportions of males and females assigned to return for screening colonoscopy within 2 years were significantly different from each other: 72% (*n* = 191) of males and 59% (*n* = 117) of females were assigned to return for their next screening colonoscopy within 2 years (Table 1) (*p* = 0.006).

**Table 1:** Colonoscopic screening frequency interval: Recommendations by Physicians

Physician recommended screening interval (Years)	Gender		*p*‐Value
	Male *n* (%)	Female *n* (%)	
<1	91 (34.2)	45 (22.7)	0.018
1–2	100 (37.6)	72 (36.4)	
2–3	38 (14.3)	34 (17.2)	
3–5	36 (13.5)	45 (22.7)	
>5	1 (0.4)	2 (1)	
Total	266 (100)	198 (100)	

#### 3.1.2 Incidence of polyps

In screened males, of 212 polyps detected, 46% (*n* = 97) were adenomatous, and 52% of these were located in the proximal colon. In screened females, of 114 polyps detected, 43% (*n* = 49) were adenomatous, and 67% (*n* = 33) were in the proximal colon (Table 2).

**Table 2:** Pathology of polyps by gender

	Male				Female			
	Proximal *n* (%)	Distal *n* (%)	Total *n* (%)	*p*‐Value	Proximal *n* (%)	Distal *n* (%)	Total *n* (%)	*p*‐Value
Adenomatous	50 (51.5)	47 (48.5)	97 (45.8)	0.178	33 (67.3)	16 (32.7)	49 (43)	0.039
Non‐adenomatous	46 (41.8)	64 (58.2)	110 (51.9)		28 (45.9)	33 (54.1)	61 (53.5)	
Mixed features	1 (20)	4 (80)	5 (2.34)		1 (25)	3 (75)	4 (3.5)	
Total	97 (45.8)	115 (54.2)	212 (100)		62 (54.4)	52 (45.6)	114 (100)	

In males, 58% (*n* = 56) of 97 adenomatous polyps were tubular adenomas, 13% tubulovillous, 4% villous, and 1% sessile serrated. Of 110 non‐adenomatous polyps, 97% (*n* = 107) were hyperplastic. In females, of 49 adenomatous polyps, 61% (*n* = 30) were tubular adenomas, 12% tubulovillous, 4% villous, 2% villotubular, 4% sessile serrated, and 2% serrated adenomas. Of 61 non‐adenomatous polyps, 98% (*n* = 60) were hyperplastic.

In screened males, the median time to detection of any incident polyp from time of entry into the study was 2.0 years (95% CI 0.03–4.0). In screened females, it was 10.3 (95% CI 5.2–15.4) years. (Supporting Information Figure S1A). In males, the median time to detection of adenomatous polyps was 12.4 (95% CI 11.5–13.3) years and in female’s time, it was 20.2 (95% CI 18.4–26.7) years. (Supporting Information Figure S1B).

### 3.2 Incidence of CRC

**Figure 3:**
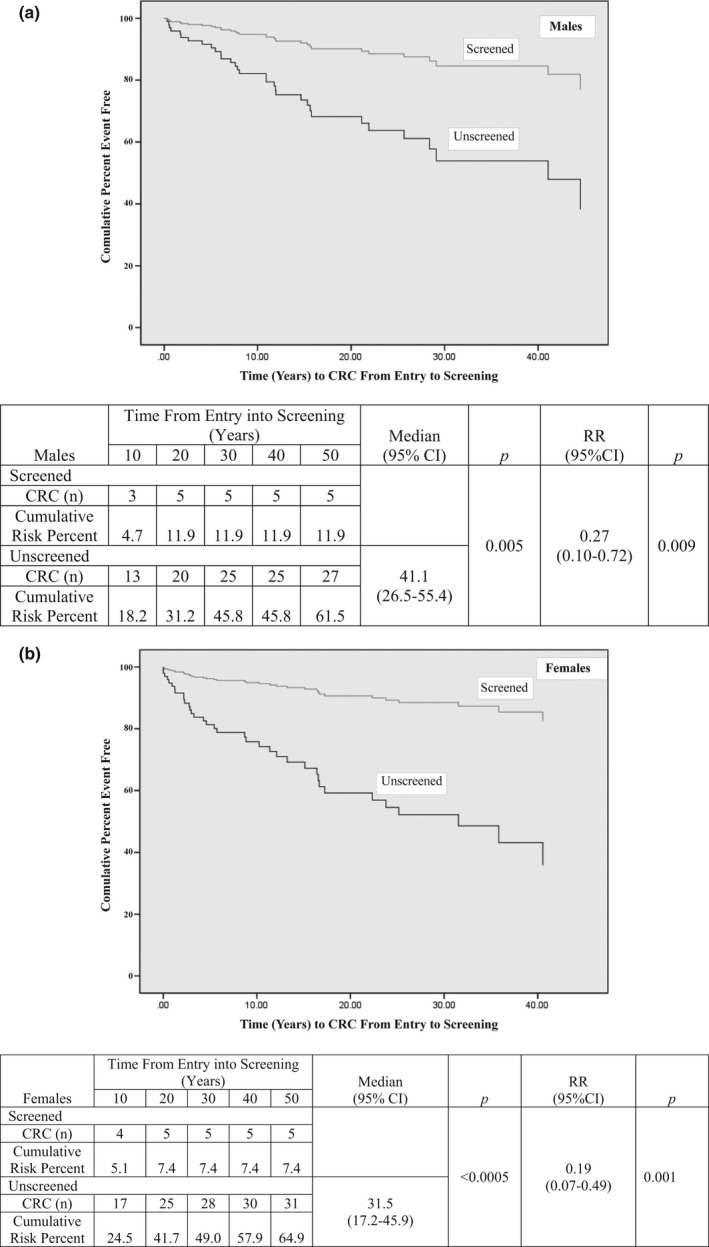
Time to CRC from entry into primary prevention screening compared to that in matched unscreened group: (a) Males; (b) Females

From time of entry into screening, 12% of males developed CRC after 30 years of follow‐up, compared to 46% of unscreened males (Relative Risk [RR] = 0.27; 95% CI: 0.10–0.71; Figure 3a). From time of entry into screening, 7% of females had developed CRC after 30 years of follow‐up, compared to 49% of unscreened females (RR = 0.19; 0.07–0.48; Figure 3b).

In the unscreened control group, there was no significant difference in time to CRC comparing those born from 1910–1949 to those born from 1950 onwards (relative risk 1.31: 95% CI 0.62–2.78).

### 3.3 Mortality

**Figure 4:**
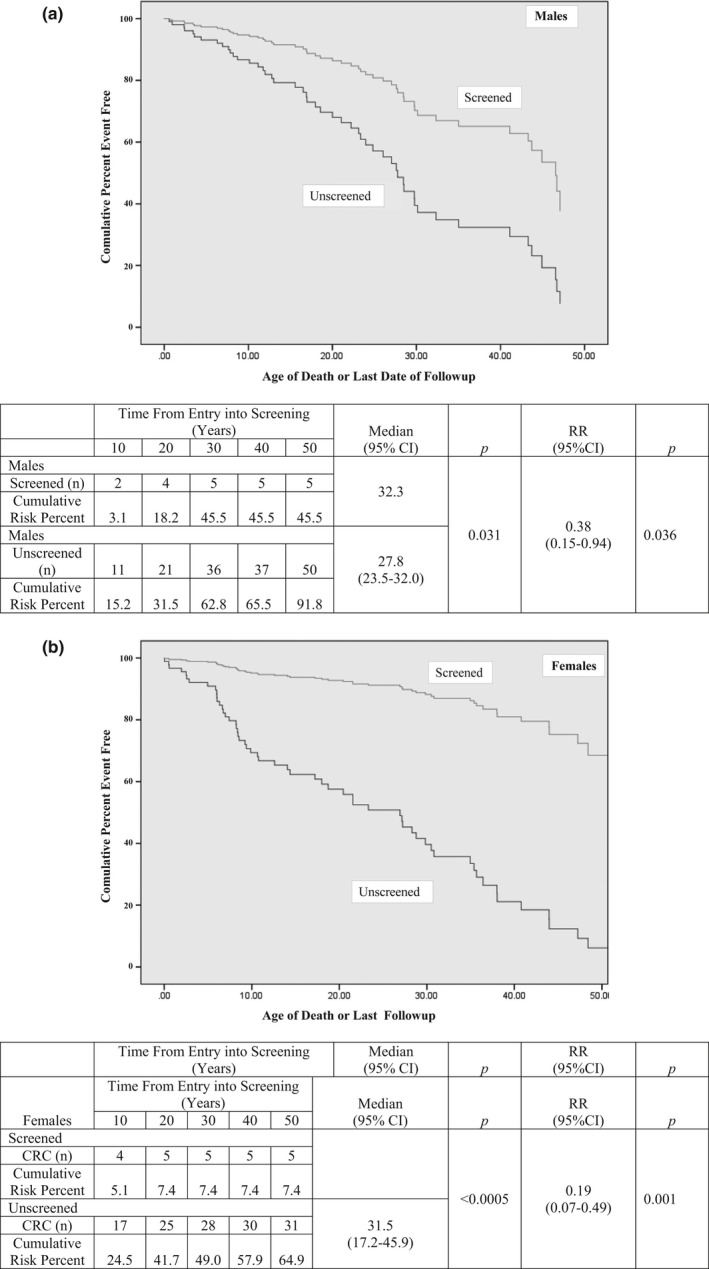
Mortality from time to entry into primary prevention screening compared to mortality in matched unscreened groups: (a) Males controls (b) Females controls

From entry into the study, survival was significantly better in screened compared to unscreened males (RR = 0.38; Figure 4a). At 30 years of follow‐up, 45.5% of males had died in the screened group compared to 62.8% in the unscreened group. In screened females, mortality at 30 years of follow‐up was 7.2%, whereas in unscreened females, it was 60.4% (RR = 0.14; Figure 4b).

The median life expectancy in screened males was 79.8 years (95% CI; 76.9–82.7), significantly better than that of unscreened males which was 75.5 (95% CI: 67.4–83.6) years. The median life expectancy could not be calculated in screened females but it was >80 years (cumulative mortality at 80 years was 34%). In unscreened females, median mortality was 73.9 (95% CI: 70.1–77.8) years. In the unscreened control group, there was no significant difference in mortality comparing those born 1910–1949 to those born from 1950 onwards (RR = 0.75: 95% CI 0.31–1.8).

### 3.4 Time from prior colonoscopy to colorectal cancer in the screened group

Of 10 CRCs in the screened group, two were diagnosed on initial colonoscopy (ages 46 and 59 years), three were diagnosed within 2 years of prior colonoscopy, and five were diagnosed >2 years after prior colonoscopy (2.2, 2.6, 3.8, 7.8 and 14.6 years).

### 3.5 Extra‐colonic cancers occurring in FCCTX families

#### 3.5.1 Non‐LS cancers

In screened males, 7 (8.5%) developed non‐LS extra‐colonic cancer compared to 11 (14.0%) in unscreened males. In screened females, 11 (13.3%) developed non‐LS extra‐colonic cancer compared to 12 (14.4%) in unscreened females. There was no difference in time to cancer comparing screened and unscreened groups.

#### 3.5.2 LS cancers

In screened males, 2 (2.5%) LS type extra‐colonic cancer diagnoses were made compared to 8 (10.1%) in unscreened males. In screened females, only one case of LS type extra‐colonic cancer was diagnosed, compared to 7 (8.4%) in unscreened females. There was no difference in time to cancer comparing screened to unscreened (Supporting Information Figure S2).

## 4 discussion

In this family‐based case–control study of primary prevention colonoscopic screening in FCCTX, the primary outcome was prevention of CRC. In the unscreened males, lifetime risk of CRC was 49% and screening colonoscopy reduced the risk of CRC by 73%. In the unscreened females, lifetime risk was 53% and screening reduced the risk by 89%. These benefits are similar to those observed in LS MMR mutation carriers where CRC risk reduction by colonoscopic screening was 71% in both males and females (Stuckless et al., 2012), using methods similar to the current study.

The CRC risk reduction in the current study was associated with significantly improved survival (RR in males = 0.38 and in females 0.14). The mortality risk reduction of colonoscopic screening in male LS MMR mutation carriers (compared to those not screened) was 0.38 (95% CI 0.13–1.0), and in female mutation carriers, it was 0.19 (0.09–0.44; Stuckless et al., 2012).

In designing this study, we were concerned about survivor bias in that only survivors without CRC could enter a screening program. Unlike in LS (Stuckless et al., 2012), family members generally entered screening before the risk period for CRC: by age 50 years, 68% of the male screened group had started screening, whereas by this age, only 9% of the unscreened group had developed CRC; for the females, the comparable proportions were 66% and 11%. This is probably not surprising seeing that median age at entry to screening was 45 years, family members were perceived to be at 50% risk of inheriting a genetic CRC susceptibility factor, and age of onset of CRC is later in FCCTX than in LS (Lindor et al., 2005).

The efficacy of screening colonoscopy is delivered via polypectomy, particularly of adenomas (Zauber et al., 2012). In the current study, 336 polyps were excised from 162 people of which 171 (51%) were adenomatous. Meta‐analysis of observational studies on screening colonoscopy in adenoma cohorts estimated the incidence of CRC was reduced by 69%, 88%; Brenner et al., 2014).

In the screened group, 10 people developed CRC: Two asymptomatic patients were diagnosed on initial colonoscopy and eight following entry into the program, two of which occurred within a year of prior colonoscopy, three after 2–4 years and two more than 5 years after prior colonoscopy. Perhaps the two CRCs occurring within a year of colonoscopy resulted from inadequate prior colonoscopy (Kaminski et al., 2010), and the two that occurred more than 5 years after colonoscopy were the result of noncompliance to screening recommendations. The three CRCs diagnosed 2–4 years following prior colonoscopy raise the question of whether the 1–2 year intervals for colonoscopy recommended in the current study or the 3–5 years recommended by guidelines is appropriate (Rex et al., 2017). The answer to this question may be informed by whether or not adenoma‐carcinoma progression is accelerated in FCCTX as it is in LS (Ahnen, 2011; Brenner et al., 2007). Although the current study provided strong evidence of the benefit of 1–2 years of screening intervals, it is possible that longer screening intervals of 3–5 years may be equally efficacious.

The limitations of the current study include non‐randomized allocation of the intervention, historical controls, retrospective data collection, and incomplete medical records. A randomized controlled trial of primary prevention screening colonoscopy would be impossible in family members with FCCTX where the lifetime risk of CRC is so high. However, analysis of the real‐life experiment undertaken in these families is a reasonable surrogate.

The fact that a majority of unscreened family members were born before 1950 compared to a minority of screened members introduces a historical control bias which does not favor the intervention when the outcome is CRC, but which favors the intervention when the outcome is death. Only family members born after 1910 were studied and the period of risk for cancer was from 1960 onwards, generally the time after the introduction of the Canadian Medicare Act in 1968, which provided universal access to hospital‐based care. The available evidence suggests an increase in incidence of CRC in NL over time (Canadian Cancer Society, 2017), and in our study, the trend was to higher incidence of CRC in controls born from 1950 onwards compared to those born from 1910 to 1949. Thus, the use of historical controls was not biased in favor of screening when the outcome was CRC. It is likely that the survival data are influenced by the historical control bias as life expectancy has increased during the last century. For 65‐year‐old Canadian men in 1970–1972, life expectancy was 78.9 years and in 2000–2002, it was 82.0. For women, comparable life expectancies were 82.7 and 85.5 years (Statistics Canada, 2018). Also, the trend in our study was to higher survival in controls born from 1950 onward compared to those born from 1910 to 1949.

Retrospective studies are poor designs to examine risk and treatment effects because of the potential for missing data. However, in this study, we focussed on hard outcome events (death and CRC incidence) and an intervention (colonoscopy) likely to be recorded in the clinical chart. Nonetheless, of 420 family members eligible for study 71 (17%) had no/incomplete health records. This likely did not favor the intervention as the majority were born from 1910 to 1950 (data not shown).

The attractions of studying outcomes of genetic diseases in Newfoundland are the existence of large families, whose members have continued to live in adjacent areas, with care provided by a small number of hospitals to a population of just over 500,000. The evaluation of the impact of primary prevention screening colonoscopy in FCCTX is thus on a background of homogenous genetic and environment influences. There likely was little exposure to referral bias as these families were identified from a 4‐year population‐based study of incident CRC patients, and from the Provincial Medical Genetics Program (Warden et al., 2013).

We conclude that a primary prevention screening colonoscopy program significantly reduced the incidence of CRC and improved survival in members of families with FCCTX, who were assumed to be at 50% a priori risk of inheriting a genetic susceptibility factor to CRC. Prospective identification of HNPCC families in the population, whether they have LS or not, allied to a primary prevention colonoscopic screening program for family members at risk of CRC, should provide meaningful clinical benefits.

## CONFLICT OF INTEREST

The authors have no conflict of interest.
