# Supplementary figures and images for: Impact of colonoscopic screening in Familial Colorectal Cancer Type X
Source: Mol Genet Genomic Med. 2018 Oct 9;6(6):1021–30. doi: 10.1002/mgg3.478 (PMC6305669; doi:10.1002/mgg3.478)

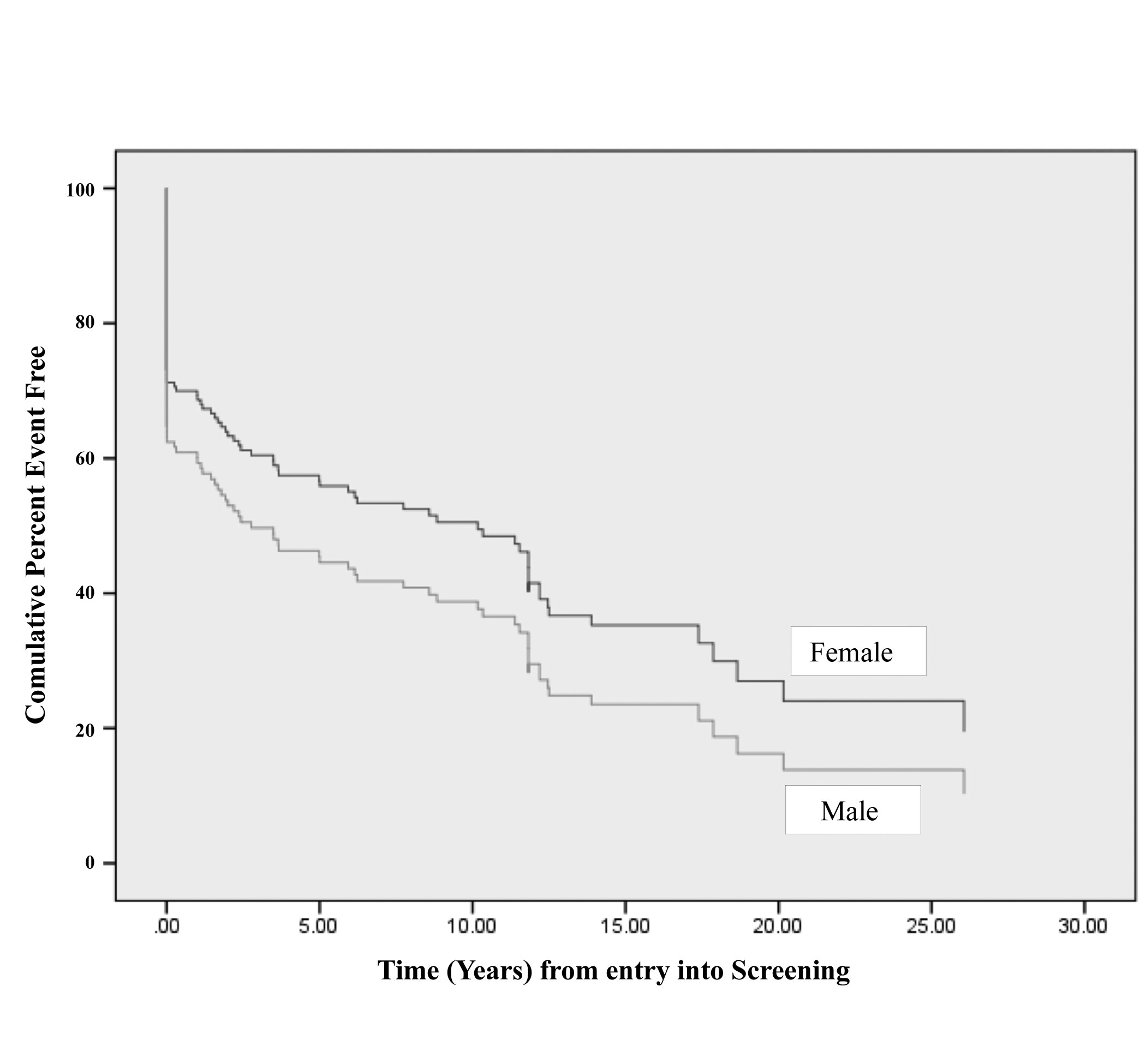

Supplement: Supplementary file 1 [file MGG3-6-1021-s001.jpg]

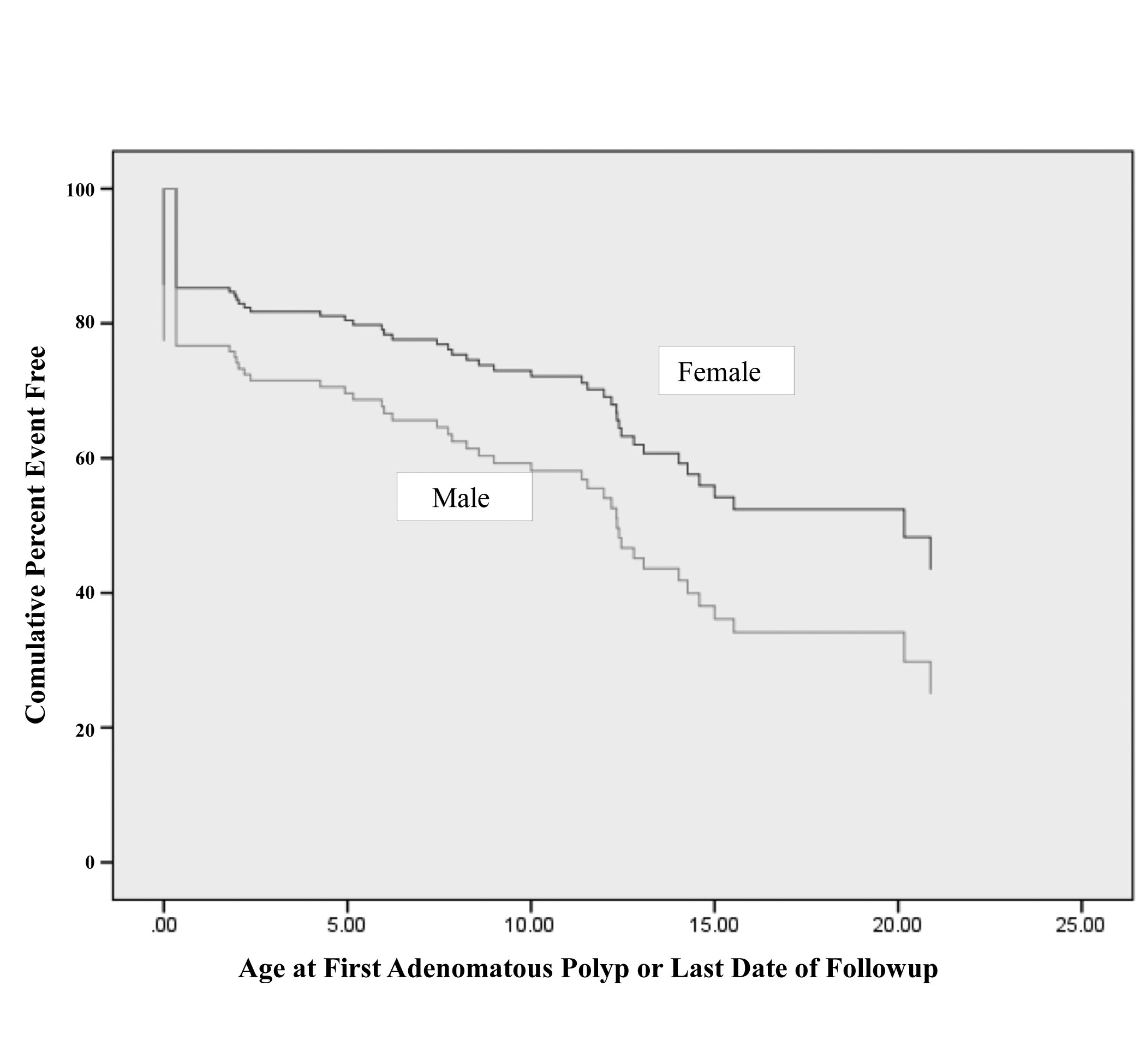

Supplement: Supplementary file 2 [file MGG3-6-1021-s002.jpg]

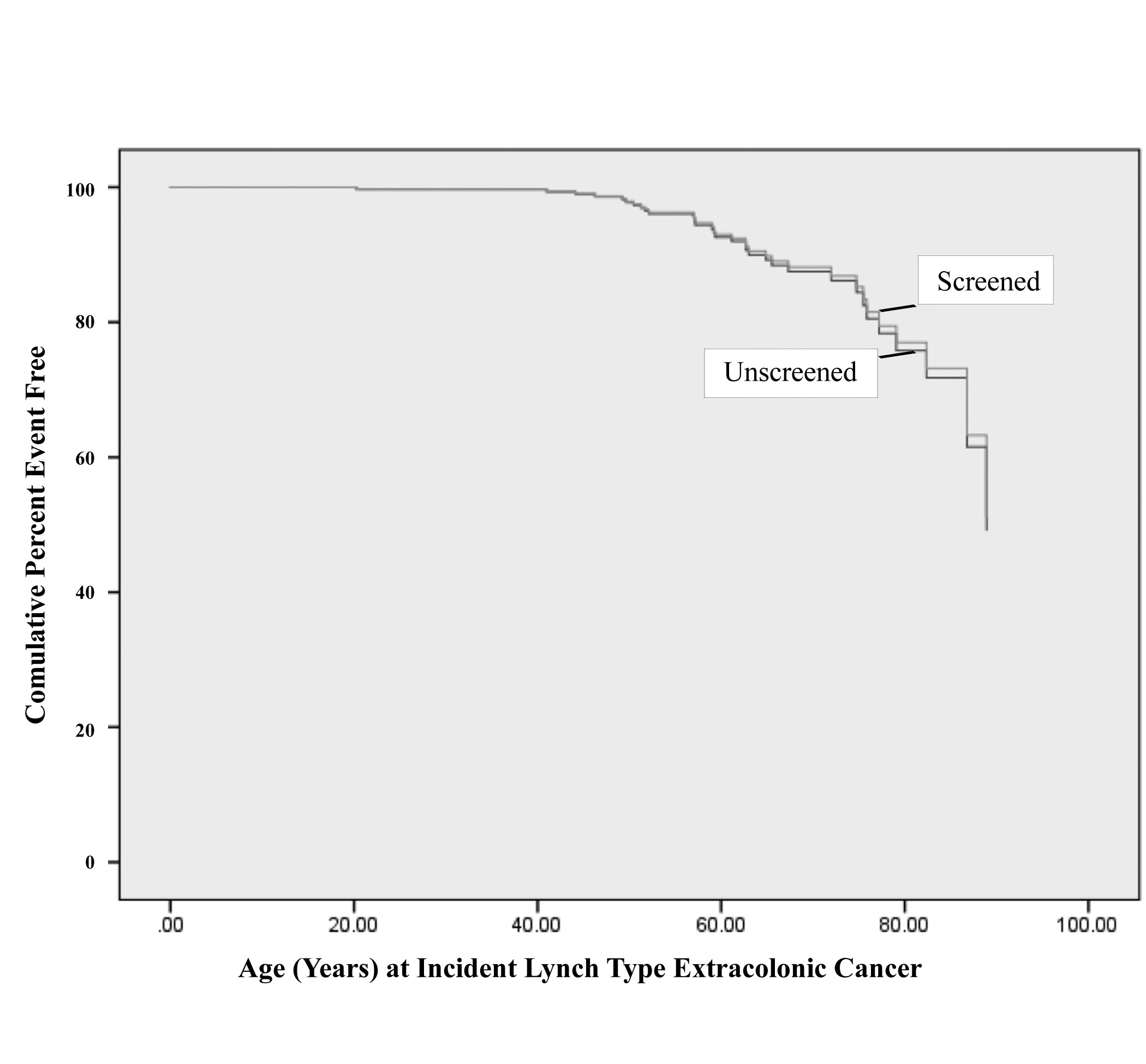

Supplement: Supplementary file 3 [file MGG3-6-1021-s003.jpg]
