# Supplementary material for: Impact of colonoscopic screening in Familial Colorectal Cancer Type X
Source: Mol Genet Genomic Med. 2018 Oct 9;6(6):1021–30. doi: 10.1002/mgg3.478 (PMC6305669; doi:10.1002/mgg3.478)
Supplement: Supplementary file 4 [file MGG3-6-1021-s004.docx]

***Suppl Figure 1A: Incidence of Any Polyp in Screened Males and Females with FCCTX from time of start of study***

***(Insert Figure here)***

| Any Incident Polyp | Time From Entry into Screening (Years) | | | | | | Median  (95% CI) | *p* | RR  (95%CI) | *p* |
| --- | --- | --- | --- | --- | --- | --- | --- | --- | --- | --- |
|  | 5 | 10 | 15 | 20 | 25 |  |  |  |  |  |
| Males | | | | | | | 2.00  (0.03-3.97) | 0.08 | 1.39  (0.93-2.08) | 0.11 |
| (n) | 41 | 43 | 49 | 50 | 50 |  |  |  |  |  |
| Cumulative Risk Percent | 55.9 | 63.2 | 77 | 82.8 | 82.8 |  |  |  |  |  |
| Females | | | | | | | 10.34  (5.24-15.43) |  |  |  |
| (n) | 30 | 35 | 42 | 44 | 45 |  |  |  |  |  |
| Cumulative Risk Percent | 39.2 | 47.7 | 63.9 | 73.7 | 79 |  |  |  |  |  |

***Suppl Figure 1B: Incidence of Adenomatous Polyps in Screened Males and Females with FCCTX from time of start of study***

***(Insert Figure here)***

|  | Time From Entry into Screening (Years) | | | | | | Median  (95% CI) | *p* | RR  (95%CI) | *p* |
| --- | --- | --- | --- | --- | --- | --- | --- | --- | --- | --- |
|  | 5 | 10 | 15 | 20 | 25 |  |  |  |  |  |
| Males | | | | | | | 20.2  (18.4 – 26.7) | 0.04 | 0.62  (0.38-1.01) | 0.05 |
| AdenoP(n) | 24 | 29 | 38 | 39 | 40 |  |  |  |  |  |
| Cumulative Risk Percent | 32 | 40.8 | 65 | 68.5 | 76.4 |  |  |  |  |  |
| Females | | | | | | | 12.4  (11.5-13.3) |  |  |  |
| Adeno P (n) | 15 | 21 | 26 | 27 | 28 |  |  |  |  |  |
| Cumulative Risk Percent | 18.6 | 29 | 41.8 | 45.5 | 53.2 |  |  |  |  |  |

***Suppl Figure 2: Cumulative incidence of LS Type Extra-colonic Cancer in both Males and Females from Birth, comparing screened to unscreened family members***

***(Insert Figure here)***

|  | Age in Years | | | | | | **Mean**  (95% CI) | *p* |
| --- | --- | --- | --- | --- | --- | --- | --- | --- |
|  | 30 | 40 | 50 | 60 | 70 | 80 |  |  |
|  | | | | | | | 80.3  (78.0-82.6) | NS |
| Screened (n) | 0 | 0 | 2 | 6 | 10 | 10 |  |  |
| Cumulative Percent | 0 | 0 | 1.7 | 7.5 | 16.6 | 16.6 |  |  |
|  | | | | | | | 85.65  (82.4-88.9) |  |
| Unscreened | 1 | 1 | 4 | 10 | 13 | 19 |  |  |
| Cumulative Percent | 0.6 | 0.6 | 2.7 | 7.6 | 10.7 | 24.3 |  |  |
